# Tear lipocalin is the major endonuclease in tears

**Published:** 2008-01-29

**Authors:** Taleh N. Yusifov, Adil R. Abduragimov, Kiran Narsinh, Oktay K. Gasymov, Ben J. Glasgow

**Affiliations:** Departments of Pathology and Laboratory Medicine and Ophthalmology, Jules Stein Eye Institute, University of California, Los Angeles, CA

## Abstract

**Purpose:**

Human endonucleases are integral to apoptosis in which unwanted or potentially harmful cells are eliminated. The rapid turnover of ocular surface epithelium and microbial colonization of the eyelids are continual sources of DNA in tears. Here, we determine the principal sources of endonuclease activity in tears.

**Methods:**

Endonucleases in human tears were identified after Sephadex G100 gel filtration. DNA hydrolyzing activity was measured by the conversion pUC19 plasmid DNA to its circular form in agarose gels. Fractions with endonuclease activity were further isolated using a combination ConA-Sepharose DNA, oligo (dT) cellulose, and anion exchange chromatographies. The molecular weights of the DNA hydrolyzing proteins were estimated in zymograms and by calibration of size exclusion chromatography. DNase activities were characterized for activity at a variety of pH and ion concentrations as well as in the presence of inhibitors including NiCl_2_, ZnCl_2_, G-actin, and aurintricarboxylic acid (ATA). To determine the mode of hydrolysis, the cleaved ends of the DNA digested by tear DNases were analyzed by 3′ and 5′ end labeling using either terminal deoxynucleotidyl transferase or polynucleotide kinase with or without pretreatment with alkaline phosphatase.

**Results:**

Tear lipocalin (TL) accounts for over 75% of the DNA catalytic activity in tears while a second endonuclease, ~34 kDa, is responsible for less than 24% of the activity. Both are Mg^2+^ dependent enzyme endonucleases that are enhanced by Ca^2+^, active at physiologic pH, inhibited by aurintricarboxylic acid, and catalyze hydrolysis of DNA to produce 3′-OH/5′P ends. However, the two enzymes can be distinguished by the inhibitory effect of NiCl_2_ and the sizes of the cleaved DNA fragments.

**Conclusions:**

Two magnesium dependent extracellular endonucleases were identified in tears that are different from other major human extracellular nucleases. TL is the principal endonuclease in human tear fluid. Tear endonucleases have unique characteristics that differ from other known human endonucleases.

## Introduction

The ocular surface of the human eye is directly exposed to many viral, bacterial, and fungal microbes but rarely becomes infected. The human tear film acts in concert with the corneal and conjunctival epithelium to protect the ocular surface. The corneal epithelium forms a barrier that is five-cell layers thick and turns over every 7–14 days [1,2]. The tear film is responsible for the clearance of DNA from both human and microbial sources. An assortment of viral nucleotide sequences have been identified in tears of patients infected with viruses including Herpes [3], EBV [4], CMV [5], RSV [6], Varicella Zoster [7,8], HIV [9], Hepatitis B virus [10], Hepatitis C virus [11], SARS [12], and adenovirus [13]. Adenoviral sequences have been detected by polymerase chain reactions (PCR) in tears as long as 13 years after presumed initial infection, and the evidence suggests that the virus persists as a chronic follicular conjunctivitis [13]. Some viruses such as HIV may be easily cultured from the blood but can not be cultured from tears, even in infected patients. Extracellular endonucleases have a potentially important role in tears for the destruction of DNA in apoptosis and the prevention of transfection of viruses to other cells.

Lipocalins, including tear lipocalin (TL), are known to have endonuclease activity in vitro, but whole tears have not been studied. The enzymatic activity of lipocalins is conferred by a conserved LEDFXR domain of the *Serratia marcescens* Mg^2+^-dependent nucleases [14]. Catalysis of DNA by TL is probably related to the magnesium water cluster formed by the hydrogen bond created between Glu-127 in a conserved α-helical segment and water. The nonspecific endonuclease activity of lipocalin is also divalent cation dependent [14]. The specific activity of TL is three orders of magnitude less than DNase I [14]. This paucity of specific activity prompted us to consider the possibility of other endonucleases in tears and determine the contribution of TL to overall enzyme activity. Further, the mode of DNA cleavage by TL is unknown but may be functionally important. DNA hydrolysis may result in either 3′-OH/5′-P or 3′-P/5′-OH ends. Here, we preliminarily characterize the influence of ions on the principal nucleases and establish the mode of DNA hydrolysis.

## Methods

### Tear collection

Tear secretion was stimulated with onion vapors, and tears were collected from the lower conjunctival cul-de-sac from healthy human donors as previously described [15].

**Figure 1:**
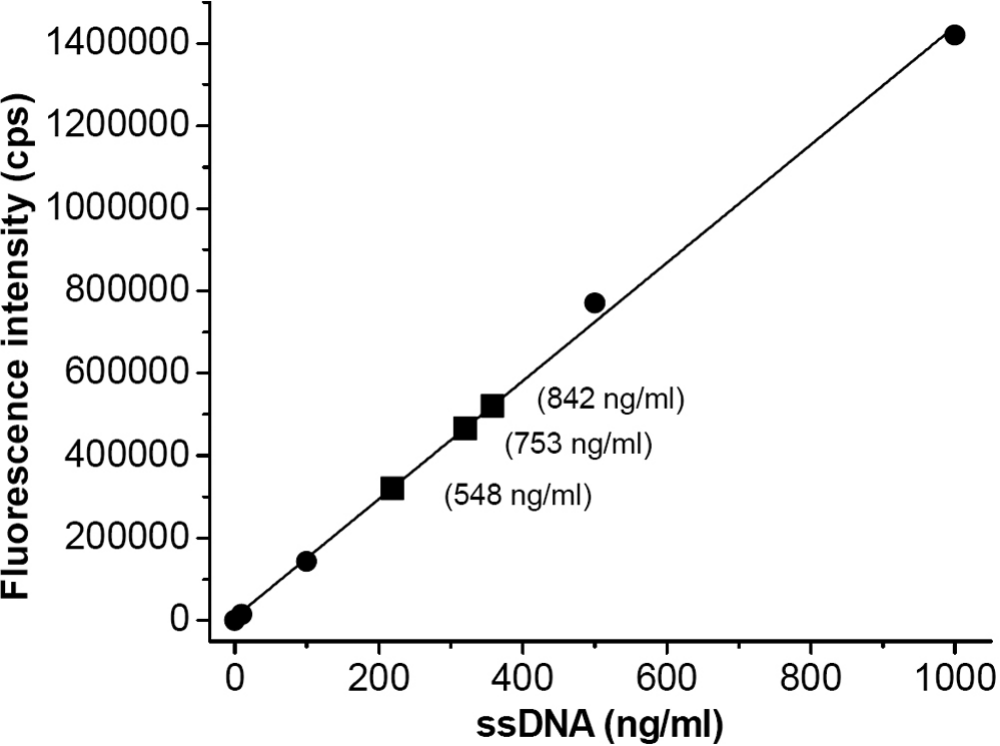
Quantitation of DNA in human tears. The oligogreen dye assay provides a measurement of the amount of DNA in freshly collected tears. The fluorescence enhancement of the dye bound to DNA is measured, λex=480 nm and λem=520 nm. The results for tear samples (■, diluted 2.125X) are plotted on a standard curve of oligonucleotides (●) of known concentration. The numbers in parentheses are the concentrations of the oligonucleotides in tears corrected for dilution.

### Quantitation of DNA in tears

Tears (0.8 ml) were collected from three individual donors and immediately treated with proteinase K, extracted with phenol:chloroform (1:1), precipitated with ethanol, and resuspended in 10 mM Tris-HCl, 1 mM EDTA, pH 7.5 [16]. The isolated DNA was quantified by a fluorescence assay (Oligreen DNA quantitation Kit, Molecular Probes, Eugene, OR). The amount of DNA in tears was determined by extrapolation from a standard curve of a serially diluted 18-mer M13 primer solution (100 μg/ml) in 10 mM Tris-HCl, 1 mM EDTA, pH 7.5. Tears were diluted 2.125 fold in the assay mixture. Steady-state fluorescence measurements were taken with a Jobin Yvon-SPEX (Edison, NJ) Fluorolog tau-3 spectrofluorometer, λ_ex_=480 nm and λ_em_=520 nm with 2 nm band widths for both excitation and emission. For each measurement, correction was made for the intrinsic fluorescence of the dye.

**Figure 2:**
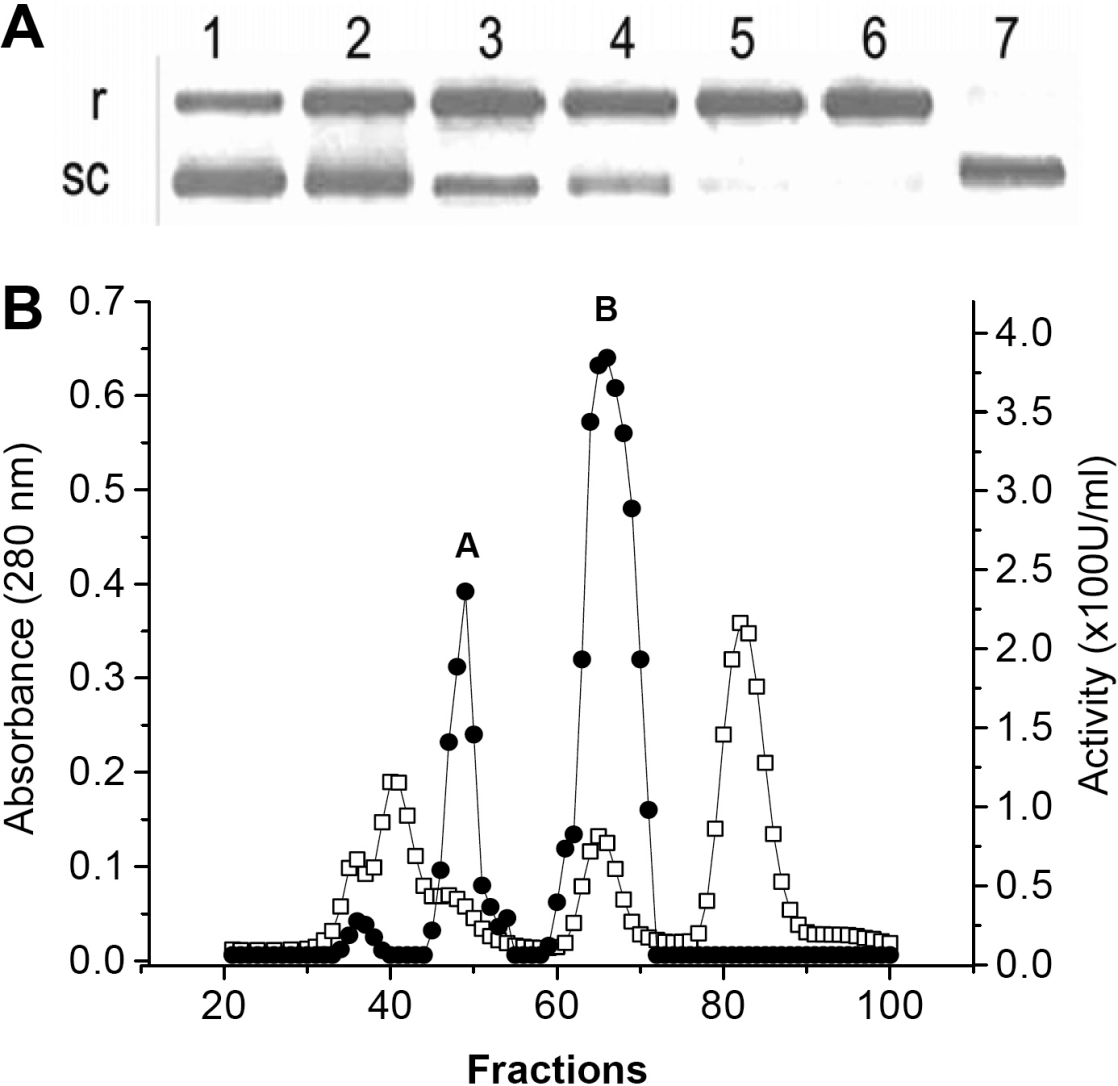
Endonuclease activity in gel filtration fractions of human tears. **A** shows the agarose gel of products from hydrolysis of pUC19 human tears (diluted 1:5) Aliquots were removed from the reaction mixture at successive 15-min. intervals (Lanes 1–6); Lane 7 shows sc pUC19 incubated only. The forms of the plasmid are indicated as relaxed (r) and supercoiled (sc). **B **demonstrates endonuclease activity profile of fractions superimposed on the absorbance elution profile from filtration of human tears (2.5 ml) on Sephadex G-100 column. (□) stands for absorbance; (●) stands for DNA hydrolyzing activity profile calculated by the densitometry of bands in the agarose gel electrophoresis of plasmid DNA sc pUC19. Each point represents the activity resulting from incubation of 5 μl of the various fractions with the plasmid.

### Endonuclease activity assay

In general, DNA-hydrolyzing activity was determined in 20 mM Tris-HCl, pH 7.5, 1 mM MgCl_2_, 1mM CaCl_2,_ 50 mM NaCl, and 0.1 μg sc pUC19 plasmid DNA (New England Biolabs, Beverly, MA) in a volume of 20 μl. For experiments investigating activity with varying ions, the other components of the assay buffer are specifically indicated. After 60 min, reaction products were separated by 0.8% agarose gel electrophoresis. Ethidium-bromide stained gels were photographed and scanned with a densitometer (IS-100 Digital Imaging System; Alpha Innotech Corporation, San Leandro, CA). To compare activities, the relative intensity of the appearance of the relaxed form of the pUC19 plasmid was quantified [14]. One unit of nuclease activity was defined as the amount needed to convert 0.1 μg sc pUC19 plasmid into its circular form after 1 h at 37 °C. To study the effects of pH on nuclease activity, the following buffers were used: sodium acetate (pH 4.0- 5,5), Mes-NaOH (pH 5.0–6.5), Mops-NaOH (pH 6.4–7.6), and Tris-HCl (pH 7.0–9.0). The nuclease activity that was observed with chemical inhibitors is presented as a percentage of the activity observed in the absence of inhibitors. For endonuclease assays of tear components, plasmid DNA was isolated and extracted with phenol:chloroform (1:1) followed by chloroform [16].

### Separation of tear components with endonuclease activity

Gel filtration chromatography was performed on 2.5 ml of pooled tears using Sephadex G-100 and eluted in 50 mM Tris-HCl, pH 8.4. Nuclease activity of whole tears was compared to that in gel filtration fractions of the same lot of pooled tears. The fractions containing the major peak of activity were analyzed by SDS tricine gel electrophoresis and further purified as previously described for TL [15]. Fractions from a second minor peak of activity partially coincided with protein peaks containing lactoferrin and lysozyme. These fractions were combined and applied to a ConA-Sepharose column (Amersham Pharmacia Biotech AB, Uppsala, Sweden) in a solution of 20 mM Tris-HCl, 50 mM NaCl, pH 7.5 to remove the lactoferrin. The flow-through fractions containing nuclease activity were isolated. Lactoferrin was eluted with a gradient of D-glucopyranoside. The fractions containing endonucleases were pooled and applied to a column of DEAE-Sephadex using the parameters as previously published for TL [15]. Eluted fractions from ion exchange chromatography with endonuclease activity were combined and applied to an oligo (dT) –cellulose column, which had been equilibrated with 50 mM sodium acetate, pH 5.2, 5% (v/v) glycerol, and 1 mM β-mercaptoethanol. The fractions were eluted with a linear gradient of NaCl (0–0.5M) in 20 mM Tris HCl, pH 7.4, 1 mM MgCl_2_, and 1 mM β-mercaptoethanol. A nuclease active fraction eluted at 75–150 mM NaCl and was concentrated centrifugally, Centricon-10 (Amicon, Bedford, MA).

### Zymographic assay and silver staining

Zymographic assays were performed in SDS/10% (w/v) polyacrylamide gels containing 10 μg/ml of heat-denatured salmon sperm DNA as previously described [14,17]. The gels were stained with ethidium-bromide. Nuclease activity appeared as clear areas against a brightly stained background of DNA. The gels were washed in 5% SDS for 1 h and silver stained, which darkened the background of the gel [18].

### End labeling reactions

pUC19 plasmid DNA was digested either by TL or the minor endonuclease followed by 3^’^-and 5^’^-end-labeling. The 3^’^-ends of the DNA fragments were labeled by [α−^32^P] dCTP with terminal deoxynucleotidyl transferase, (Fisher Scientific, Tustin, CA) and the 5^’^-ends were labeled by [γ-^32^P] ATP with polynucleotide kinase [16]. The phosphoryl groups at the ends of DNA chains were removed by pretreatment of calf intestine alkaline phosphatase (Fisher Scientific) [16,19].

**Table 1:** Nuclease activity in gel filtration fractions of human tears

Sample	Nuclease Activity (Units)	Protein (mg)
Tears	3600	7.2
Minor endonuclease (peak A)	680 (23%)	0.41
Tear lipocalin(peak B)	2162 (75%)	1.2

Peaks A and B refer to peaks of nuclease activity observed in Figure 2B. The percentages of total activity are shown in parentheses.

### Hydrolysis of single- and double-stranded DNA substrates

A 211-base pair, double stranded (ds) DNA substrate was amplified using the plasmid, pET (P20), as the template and a 5′-radiolabeled primer that had been previously reacted with [γ-^32^P] ATP and T4 polynucleotide kinase. After agarose gel electrophoresis, the PCR product was eluted and additionally purified using QIA-Quick spin columns (Qiagen Inc., Valencia, CA). For a single stranded (ss) DNA substrate, the 32-mer oligodeoxynucleotide, TCC AAA TGA GTA CCT GGG GGG ACT TAG GAC CT (Invitrogen, San Diego, CA), was eluted from a 25% polyacrylamide gel and labeled at the 5′ end through treatment with [γ-^32^P] ATP and T4 polynucleotide kinase. Both single- and double-stranded DNA templates were subjected to nuclease digestion by TL and the minor tear endonuclease. DNase I was used as a control. The cleavage reactions were performed with 30–50 nM DNA and 10 U nuclease containing 20 mM Tris-HCl, pH 7.5, 1 mM MgCl_2_, 1mM CaCl_2,_ and 50 mM NaCl. Aliquots were sampled at different time intervals and analyzed by electrophoresis. Cleavage products of dsDNA were run on the denaturing 6% polyacrylamide gel using the products from a sequencing reaction of the same DNA fragment to estimate the size of the fragments. ssDNA digestion products were analyzed in a denaturing 20% polyacrylamide gel.

## Results and Discussion

### DNA in tears

Human, viral, and bacterial DNA can be expected in tears. The quantitation of oligonucleotide DNA in tears from a standard curve using oligogreen dye is shown in Figure 1. After the correction for dilution, the final concentration of oligonucleotides found in human tear samples was 714±151 (standard deviation) nanograms/ml. Calculations from the shedding rates of the epithelial cell turnover suggest a contribution from the cornea of about 500 ng/ml DNA in tears [1,2]. The range of values measured for oligonucleotides in tears, 548–842 ng/ml, appears roughly concordant. Both the theoretical and measured values are probably underestimated. DNA from conjunctiva sources was not included in calculations of cellular turnover. Free nucleotides may not be fully recovered from the extraction process, and stimulated tear collection inevitably results in some dilution. Given these limitations, the detection of oligonucleotides provides evidence that hydrolyzed DNA is present in tears.

**Figure 3:**
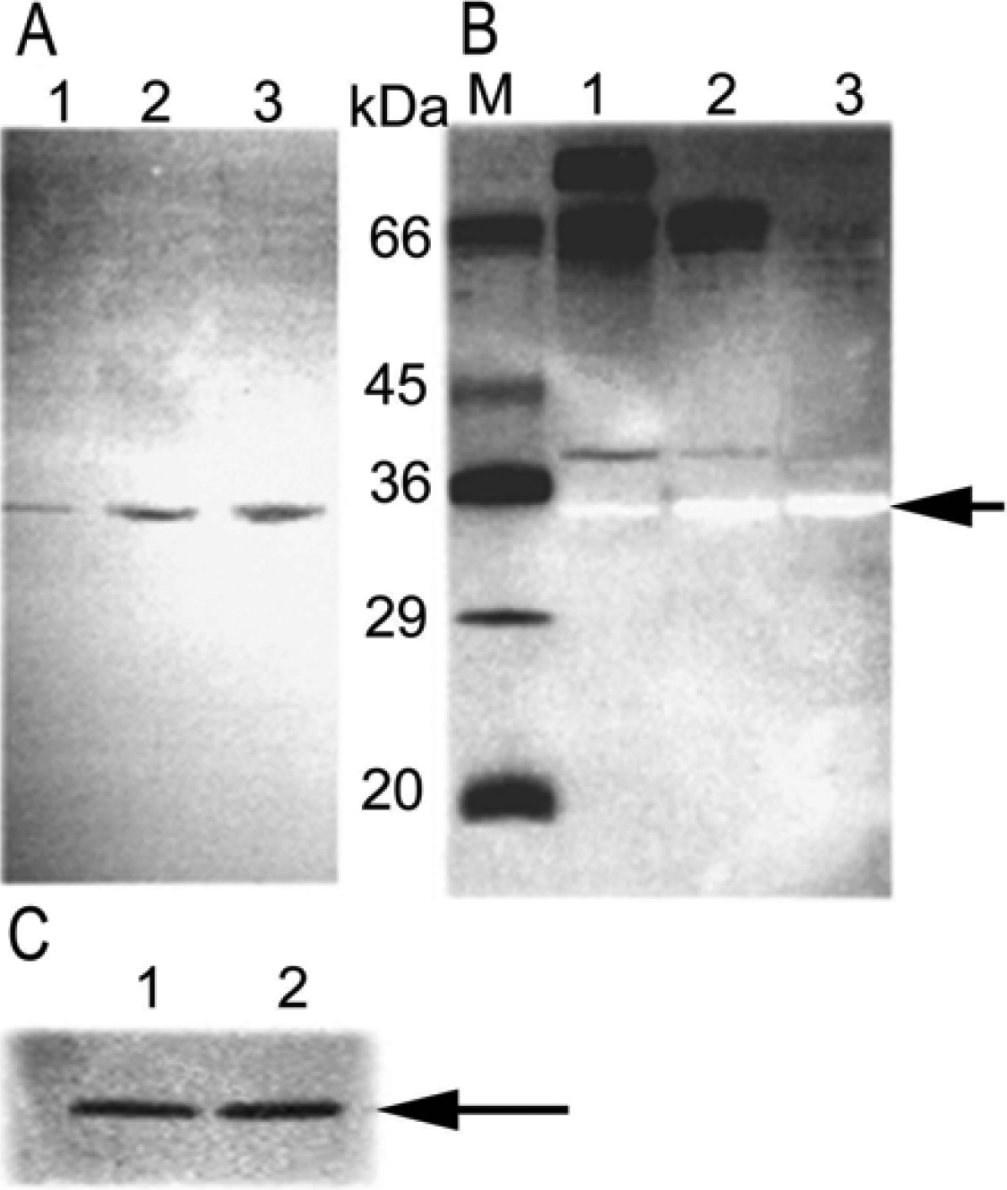
Zymographic analysis of samples after the enrichment of the minor endonuclease. **A**: The zymogram shows progressively increasing endonuclease activity: lane 1, pooled G-100 fractions “A”; lane 2, fractions with activity pooled from ConA-Sepharose; lane 3, fractions with activity pooled after oligo (dT) cellulose chromatography. **B**: Zymographic gel stained with silver. Lanes 1–3 are the same as **A**. The arrow indicates the position of the minor tear endonuclease activity. M, molecular weight markers as shown. **C**: Zymogram of the minor tear endonuclease (~34 kDa) in the presence (lane 1) and absence (lane 2) of β−mercaptoethanol.

### Endonuclease activity in human tears

DNA was hydrolyzed in human tears (Figure 2A). Two sources of endonuclease activity are evident from gel filtration chromatography (Figure 2B, designated as peaks A and B). About 80% of total endonuclease activity was recovered from gel filtrations fractions (Table 1) and over 75% of the recovered activity can be attributed to TL (peak B). A lesser peak (Figure 2B, peak A), corresponding to about 23% of the endonuclease activity, emerged in the shoulder of the absorbance peak corresponding to lactoferrin. Minimal activity, less than 2% of the total, was found in a third peak co-eluting with lactoferrin, fractions 29–39 (Figure 2B). Activity was not observed in the zymographic gel electrophoresis in this region and was deemed insignificant. The scarcity of activity of lactoferrin in tears is important since lactoferrin has DNase activity in human milk [20,21]. This result suggests a paucity of either the isoforms’ conferring activity or histidyl bound copper ions required for activity [20].

**Figure 4:**
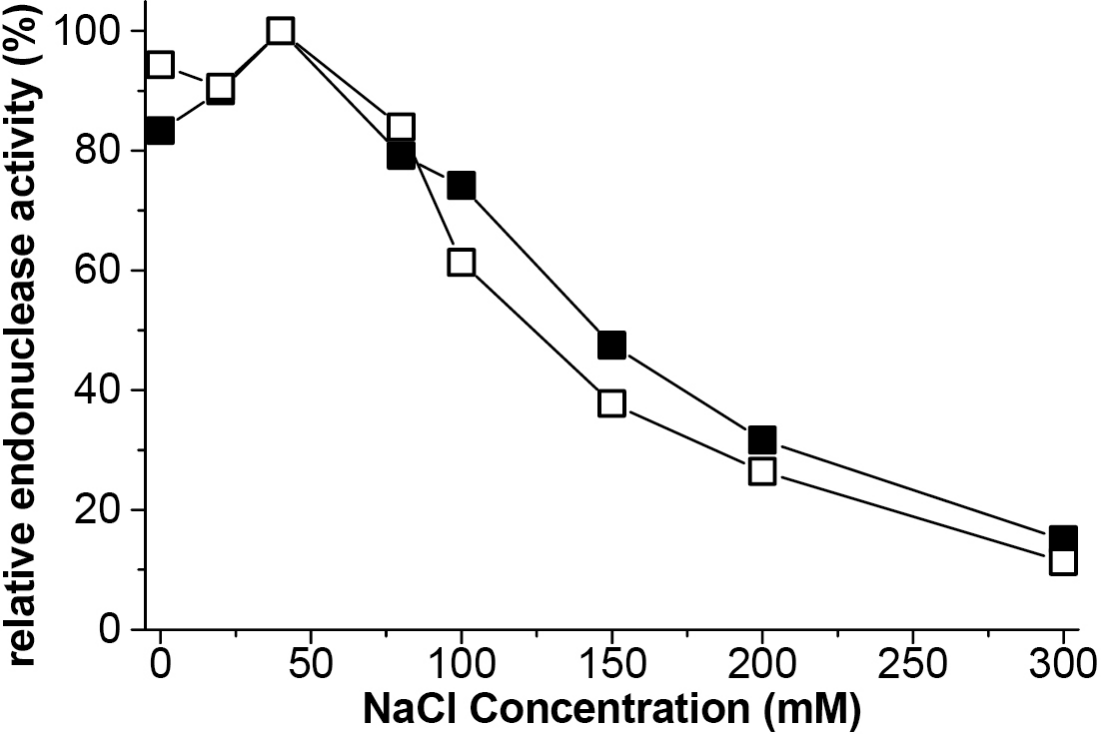
The effect of sodium chloride on human tear endonucleases. Effect of NaCl concentration on activities of TL (■) and the minor endonuclease (▲). The standard deviations of all points varied between 10%–15%. Activity for both tear endonucleases peaks at 50 mM and decreases rapidly with increasing salt concentration.

**Figure 5:**
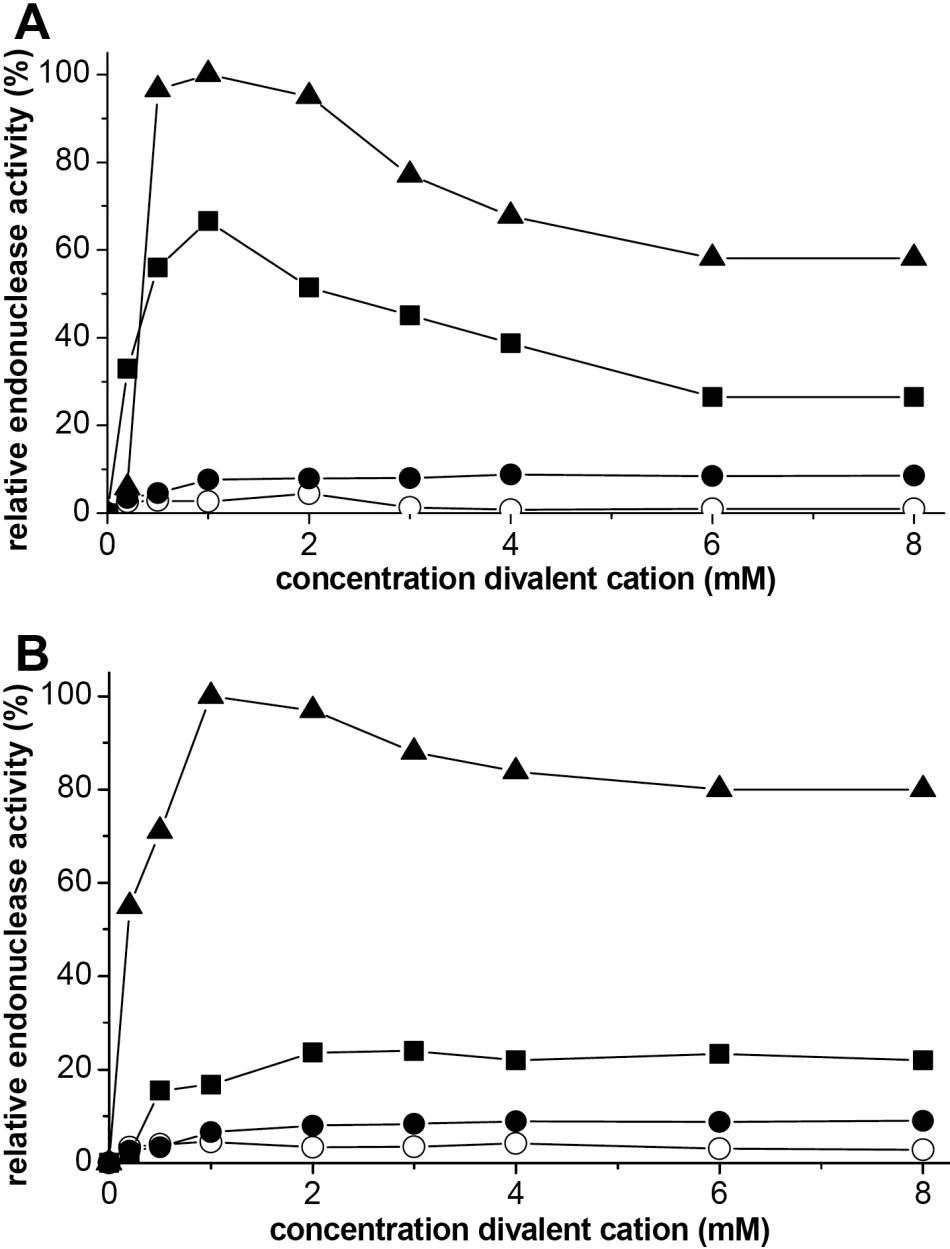
Effect of divalent cations on tear nucleases. The activities of the minor endonuclease (**A**) and TL (**B**) in the presence MgCl_2_ (■), CaCl_2_ (◯), or CoCl_2_ (●) as well as MgCl_2_. in combination with 1 mM with CaCl_2_ (▲) is shown in these graphs. Values are representative of the experiments run in triplicate. The standard deviations of all points varied between 10%–15%. Activity for both endonucleases dependent on the presence of MgCl_2_. While CaCl_2_ is not required for activity the effect of effect of MgCl_2_ and CaCl_2_ is synergistic for both endonucleases.

The zymogram that performed on the pooled fractions from minor peak A is shown in Figure 2B. A single band is evident at approximately 34 kDa (Figure 3A, lane 1). The silver stained gel reveals other proteins without nuclease activity in this sample (Figure 3B, lane 1). Lactoferrin, apparent molecular weight of about 82 kDa, was removed from the minor endonuclease fractions by chromatography with ConA-Sepharose (Figure 3B, lane 2). A 66 kDa protein remained in the sample with the minor endonuclease (Figure 3B, lane 2). Oligo-dT chromatography was used for further enrichment of the minor endonuclease fraction (Figure 3B, lane 2 and 3). Despite the enhancement of activity and clear demonstration of a band at 34 kDa in the zymogram (Figure 3A, lane 3), a protein band was not evident in the SDS-PAGE gel stained with silver (Figure 3B). Disulfide reduction did not affect the position or activity of the minor endonuclease in the zymogram (Figure 3C). TL contributes most to the endonuclease activity; the relatively minor activity from peak A is apparently derived from very little protein and therefore may have a high specific activity. Although the focus of this study was on identifying the major endonuclease in tears, we were able to conduct some preliminary enzyme characterization.

### Effect of ions on endonuclease activity

The effect of NaCl concentration was determined for both TL and the minor endonuclease at physiologic pH. Both endonucleases in tears show maximal activity in hydrolyzing plasmid DNA in 50 mM NaCl (Figure 4). DNase activity of both enzymes was abrogated in concentrations above 50 mM NaCl. At 100–130 mM NaCl (physiologic concentration, unstimulated tears), the activity of the minor endonuclease was reduced from maximal activity by about 45–55% compared to about a reduction of 25–40% for TL (Figure 4). The sodium concentration of normal tears ranges from 80-180 mM [22]. Endonuclease activity drops rapidly with increasing concentration so that in the middle of the physiologic range, the activity is reduced to about 50% of maximal activity. Stimulated tears, presumably dilute, may have more endonuclease activity while tears of dry eye patients with a higher osmolality may have reduced activity.

**Figure 6:**
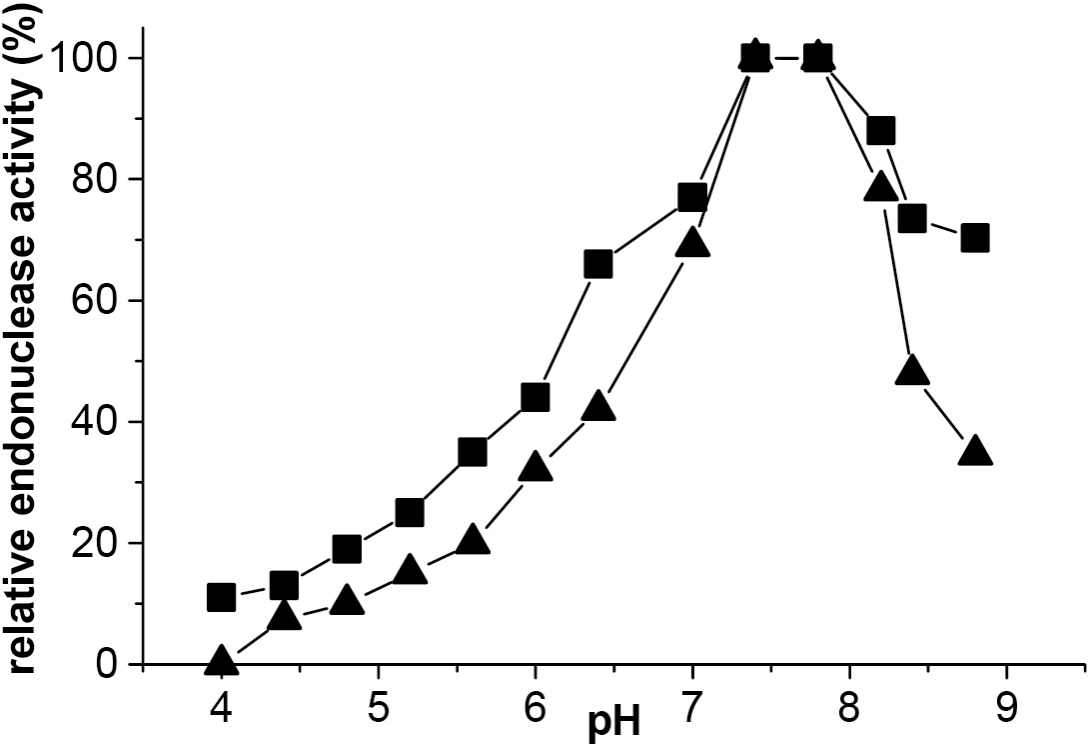
Human tear endonucleases are active at physiologic pH. Effects of pH on nuclease activity for the minor endonuclease (■) and TL (▲).The standard deviations of all points varied between 10%–15%. Both human tear endonucleases have greatest activity at physiologic pH. The activity decreases rapidly outside the physiologic range.

The divalent cation requirements for the tear nucleases were next examined. Both TL and the minor endonuclease are Mg^2+^ dependent nucleases (Figure 5). There is scarce activity in the presence of Ca^2+^ alone. The effect of Mg^2+^ is enhanced in the presence of Ca^2+^ for both nucleases. Neither enzyme was affected by Co^2+^ concentration (Figure 5).

**Figure 7:**
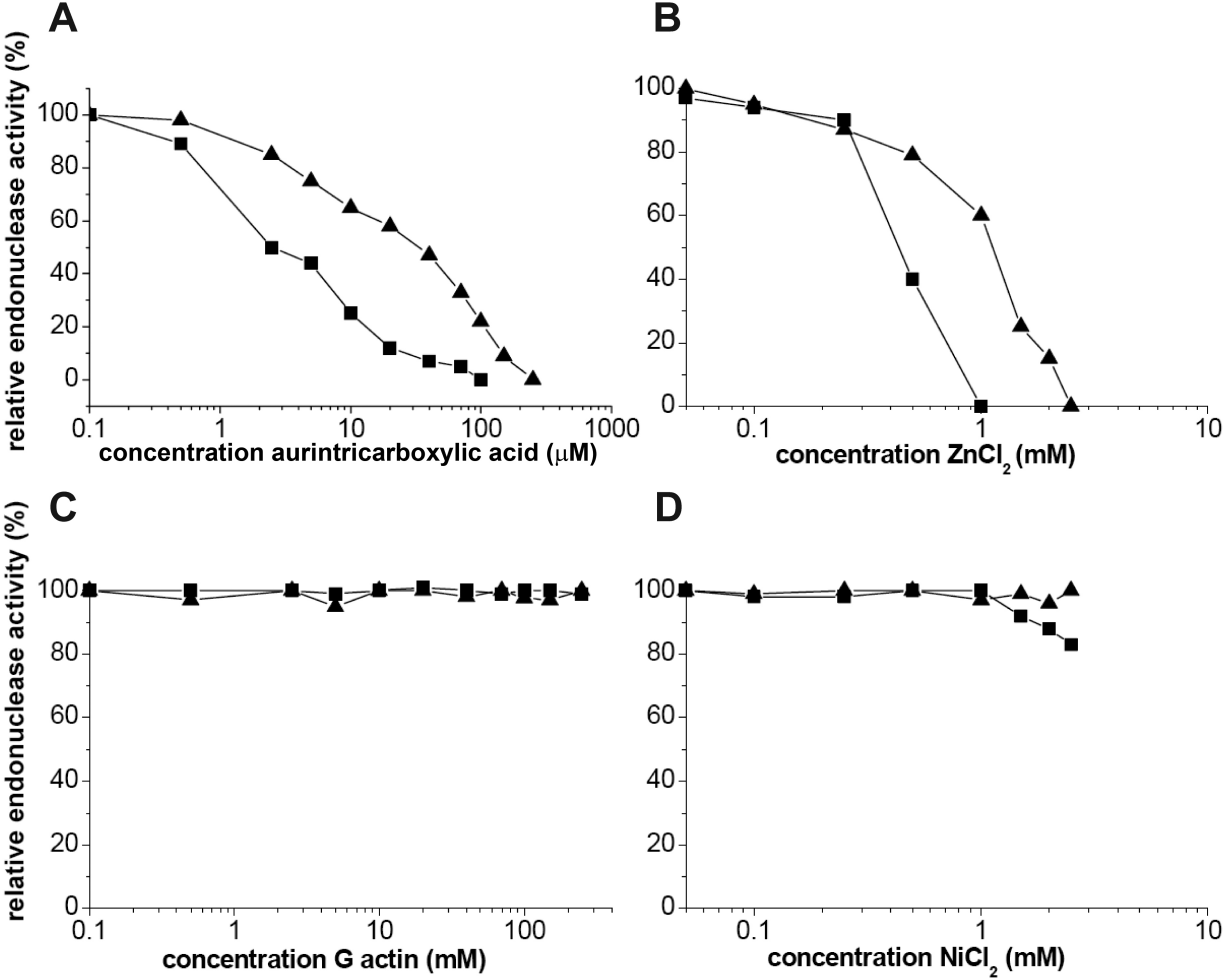
Characterization of tear endonuclease activity with inhibitors. Effects of endonuclease enzyme inhibitors: aurintricarboxylic acid (**A**), ZnCl_2_ (**B**), G-actin (**C**) and NiCl_2_ (**D**) on the activity of the minor endonuclease (■) and TL (▲).The standard deviations of all points varied between 10%–15%. Unlike other DNAses, the tear endonucleases are both sensitive to aurintricarboxylic acid and ZnCl_2_, but a different concentrations. Neither was affected by G-actin. The activity of TL was not abrogated by NiCl_2_. The minor endonuclease was only inhibited at a concentration of NiCl_2_ greater than 1 mM.

The enzymatic behavior of TL and the minor endonuclease differ from other DNases with varying concentrations of divalent cations [23]. Neither of the two endonucleases in tears is activated solely by Ca^2+^, excluding a significant contribution from the major human extracellular endonucleases, DNase I and DNase X. Both of these are activated by either Ca^2+^ or Mg^2+^ alone. DNase I is found in many body fluids and plays a role in eliminating extracellular DNA in humans [24]. DNase I deficient mice result in the appearance of an autoimmune disease similar to systemic lupus erythematosus [25]. DNase X is a glycosyl phosphatidyl inositol anchored membrane protein located on the cell surface and may prevent endocytosis-mediated transfer of foreign DNA in human muscle [26]. The minor tear endonuclease is active with Mg^2+^ alone. This is different from two human intracellular DNase γ and DNAS1L2, which require both Ca^2+^ and Mg^2+^. DNase γ is found in the spleen, liver, and bone marrow [27]. DNase γ is perinuclear and translocates to the nucleus during apoptosis [28]. Similar to the DNase I family of DNases, TL and the minor endonuclease are synergistically activated by Ca^2+^ and Mg^2+^ (Figures 5A,B).

Both TL and the minor endonuclease are inhibited by low concentrations of aurintricarboxylic acid, Zn^2+^, and Co^2+^, dissimilar to DNase γ. Like DNAS1L2, Ni^2+^ minimally affected the activity of the minor endonuclease or TL [23]. The characteristics of the tear endonucleases appear unique compared to other known human endonucleases. A bacterial source of the minor endonuclease cannot be excluded since tears are in contact with eyelids that are colonized by a wide assortment of bacteria.

**Figure 8:**
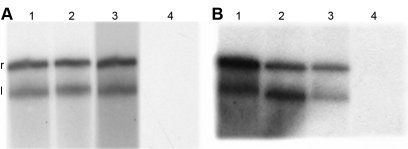
Labeling of the fragments DNA digested by tear lipocalin and the minor tear endonuclease. Kinetics of nicking plasmid DNA by nucleases, TL and minor endonuclease, were obtained as described under Methods. Aliquots were taken in different time intervals and supplemented with 2 U DNA-polymerase I for 5 min at room temperature. Heating at 70^◦^C for 5 min terminated the reaction. The amount [α-^32^P] DTP incorporated was calculated for each point time. Plasmid DNA digested by TL (**A**) or the minor endonuclease (**B**) were subjected to 3^’^ (lanes 1, 3) or 5^’^ (lanes 2, 4) end-labeling with (lanes 1, 2) or without (lanes 3, 4) pretreatment with alkaline phosphatase. Plasmid DNA was separated by 0.8% agarose gel electrophoresis and analyzed by autoradiography. The forms of plasmid DNA are indicated to the left of the panel: relaxed (r), linear (l).

DNase activity was sensitive to pH for both enzymes; the greatest endonuclease activity was observed in the range of pH 7.2–7.6 (Figure 6). Both showed some activity at acidic pH. The minor endonuclease appeared to retain slightly more activity than TL outside the physiologic pH range of tears (~7.2–7.8) [22]. Beyond this range, the activity falls precipitously, especially at acidic pH. This behavior is similar to other human endonucleases with one exception [23]. DNAS1L2 has been observed to have maximal activity at pH 5.6 [23], excluding it as a candidate for the minor endonuclease. DNAS1L2 is considered an acid endonuclease and may have a role in inflammation, but little is known about this enzyme [29].

The effects of known DNase inhibitors on TL and the minor endonuclease are shown in Figure 7. Both were inhibited by increasing concentrations of aurintricarboxylic acid (Figure 7A) and Zn^2+^ (Figure 7B), but neither was affected by G-actin (Figure 7C). There were minimal inhibition of TL by Ni^2+^ and a loss of about 20% activity of the minor endonuclease at 3 mM Ni^2+^ (Figure 7D). The lack of activity with Ca^2+^ and Co^2+^ as well as the lack of inhibitory effect by G actin for both TL and the minor endonuclease contrast with what occurs for DNase I and DNase X.

**Figure 9:**
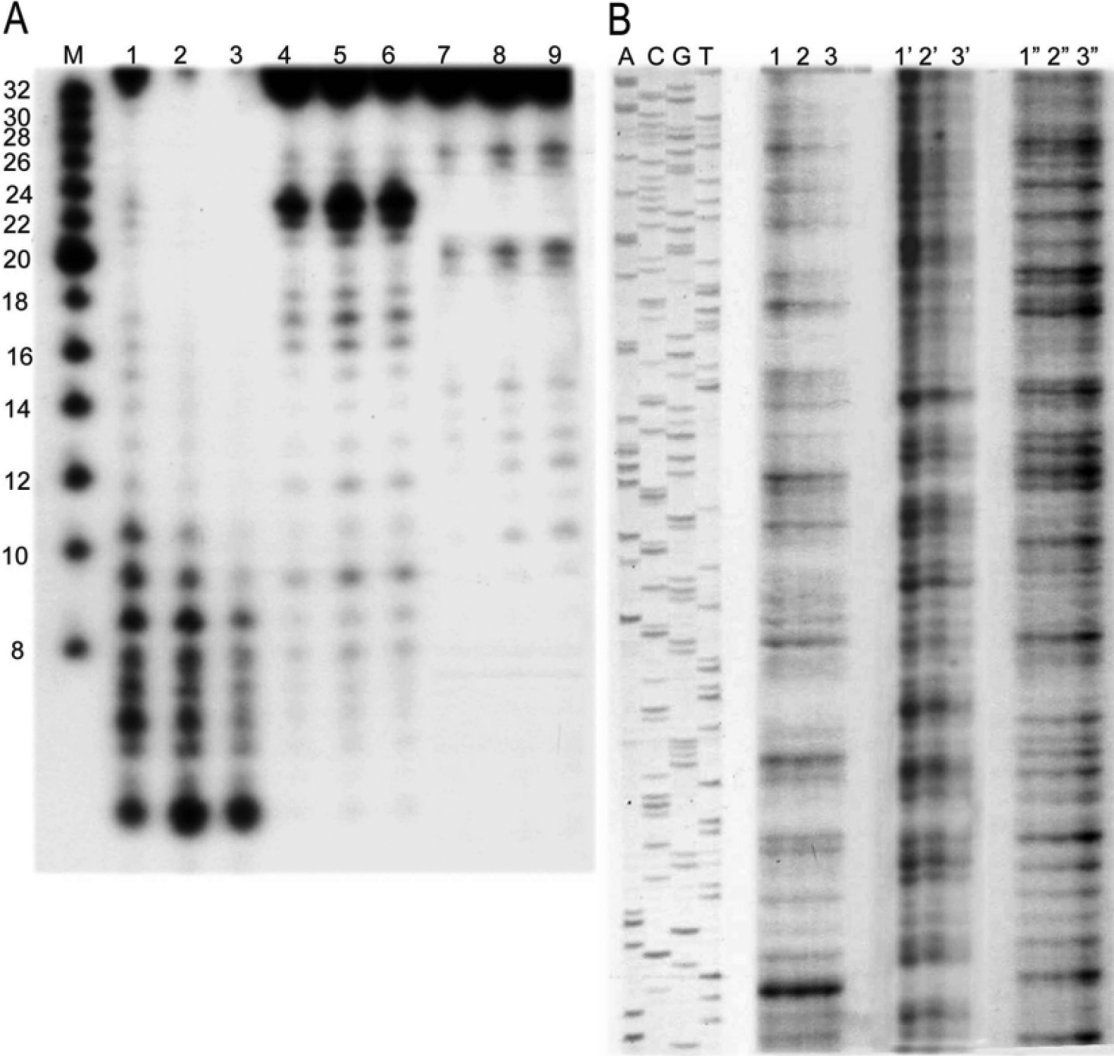
Cleavage of a radioactively labeled single- and double-stranded DNA by tear lipocalin and EndoA. **A**: A 32-mer oligonucleotide was digested by TL (lanes 1–3), the minor tear endonuclease (lanes 5–7), or DNase I (lanes 6–9) at 5, 10, and 20 min, respectively. The lane designated M shows oligonucleotide sizing markers. The size of the single strand digestion products differ for the three enzymes. **B**: A 211 bp PCR product was digested by TL (lanes 1, 2, 3), DNase I (lanes 1’, 2’, 3’), or the minor endonuclease (lanes 1”, 2”, 3”) in 10, 20, and 30 min, respectively. The lane headings, A, T, G, and C, identify the respective base-specific sequencing reactions. Sequencing was performed using the dideoxy method with T7 DNA-polymerase. The corresponding sequence of the dsDNA is shown (left). The varying intensity of the bands at the sequences shown reflect different rates of cleavage related to relative sequence preferences for each of the three enzymes.

### Mode of DNA hydrolysis

TL and the minor endonuclease catalyze DNA hydrolysis to produce 3′-OH/5′-P ends, similar to all of the members of the DNase I family. As shown in Figure 8, the 3` ends of the products of both TL (panel A) and the minor endonuclease (panel B) were labeled regardless of alkaline phosphatase pretreatment. In contrast, the 5`-ends could be labeled only after the removal of the phosphoryl groups, indicating that both TL and the minor endonuclease catalyze DNA hydrolysis to produce 3`-OH/5`-P ends.

To compare the DNA fragment sizes remaining after hydrolysis by TL and the minor endonuclease, dideoxy sequencing gel electrophoresis was performed on the digestion products (Figure 9). For single strand hydrolysis (Figure 9A), the majority of the digestion products were less than 8 bp and 26 bp with TL and the minor endonuclease, respectively. The size variation of the final single strand hydrolysis products observed in sequencing gels for DNase I, TL, and the minor endonuclease may reflect mechanistic interactions of the DNA substrate with unique binding and cleavage sites. The electrophoretic cleavage patterns in the double strand hydrolysis (Figure 9B) indicate sequence preferences for the three enzymes. Nonspecific nucleases cleave at nearly all positions but will exhibit variations in cleavage rates depending on the DNA sequence. The sequence dependent rate variation results from many local and global factors including the DNA geometry, flexibility, and bending [30]. The hydrolysis pattern with TL (Figure 9B, lanes 1, 2, and 3) features larger gaps compared to DNase I and the minor endonuclease. In addition, an intense signal in one of the smaller fragments was not observed with the other enzymes. The different sequence preferences for all three enzymes undoubtedly reflect different active sites. Although the structure of TL has been solved [31,32], a more thorough understanding of sequence cleavage preferences awaits structural analysis of the enzyme-oligonucleotide complexes.

### Functional significance of tear endonucleases

Clearance of human DNA from apoptotic cells is an obviously important potential function for nucleases in tears, but presumably breakdown is at least initiated by intracellular mechanisms.

The effective clearance of viral DNA from tears may require an extracellular endonuclease. Adenovirus infection of the conjunctiva and corneal epithelium may persist for years after infection [13]. Adenoviral DNA sequences may be amplified from tears. Some viruses such as HSV-1 suppress cell apoptotic fragmentation of DNA by evading the action of intracellular caspases and the activation of p38 mitogen activated protein kinase (MAPK) [1,33,34]. The action promotes the propagation of infection of other cells by promoting replication, transformation, and survival of the virus. A mechanism for extracellular DNA catalysis could counter viral propagation by destroying the viral DNA outside of the intracellular caspase mechanism. Transformation of viral DNA occurs readily in human corneal cells in vitro; adenoviruses transfer with 95% efficiency. As the major endonuclease of tears, TL may have a role in the prevention of viral propagation by transformation. The absence of the major human extracellular nucleases in tears, DNase I and DNase X, serves to heighten the importance of TL in degrading foreign DNA.
